# The Influence of Air Pressure on the Dynamics of Flexural Ultrasonic Transducers

**DOI:** 10.3390/s19214710

**Published:** 2019-10-30

**Authors:** Andrew Feeney, Lei Kang, William E. Somerset, Steve Dixon

**Affiliations:** Department of Physics, University of Warwick, Coventry CV4 7AL, UK; a.feeney@warwick.ac.uk (A.F.); l.kang.1@warwick.ac.uk (L.K.); w.somerset@warwick.ac.uk (W.E.S.)

**Keywords:** air-coupled ultrasound, elevated pressure, flexural ultrasonic transducer, pitch-catch ultrasound measurement, pressure measurement, unimorph transducer

## Abstract

The flexural ultrasonic transducer comprises a piezoelectric ceramic disc bonded to a membrane. The vibrations of the piezoelectric ceramic disc induce flexural modes in the membrane, producing ultrasound waves. The transducer is principally utilized for proximity or flow measurement, designed for operation at atmospheric pressure conditions. However, there is rapidly growing industrial demand for the flexural ultrasonic transducer in applications including water metering or in petrochemical plants where the pressure levels of the gas or liquid environment can approach 100 bar. In this study, characterization methods including electrical impedance analysis and pitch-catch ultrasound measurement are employed to demonstrate the dynamic performance of flexural ultrasonic transducers in air at elevated pressures approaching 100 bar. Measurement principles are discussed, in addition to modifications to the transducer design for ensuring resilience at increasing air pressure levels. The results highlight the importance of controlling the parameters of the measurement environment and show that although the conventional design of flexural ultrasonic transducer can exhibit functionality towards 100 bar, its dynamic performance is unsuitable for accurate ultrasound measurement. It is anticipated that this research will initiate new developments in ultrasound measurement systems for fluid environments at elevated pressures.

## 1. Introduction

The transmission and measurement of ultrasound in different fluid environments is vital in applications including sonar, non-destructive testing and evaluation, and flow measurement [1,2,3]. Different ultrasonic transducer designs are available which possess features suited to a range of applications or environments. For example, the flextensional configuration of an ultrasonic transducer comprises seven distinct classes [4], some of which have been utilized in applications including underwater sonar and more recently, as a transducer array for transdermal drug delivery [5]. Other forms of transducer including the piezoelectric micromachined ultrasonic transducer (PMUT) and the capacitive micromachined ultrasonic transducer (CMUT) have both been used for non-destructive testing and evaluation, ultrasound imaging, and for operation in liquids and gases [6,7,8].

**Figure 1:**
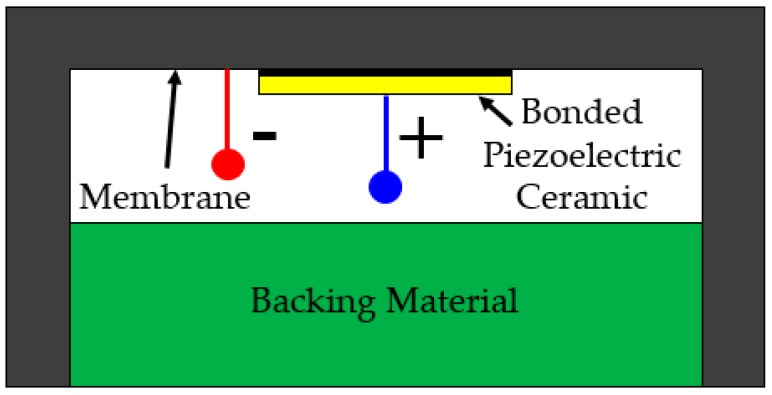
A generalized schematic of a conventional flexural ultrasonic transducer (CFUT), where the backing material is typically silicone.

A transducer type that has recently received significant attention is the flexural ultrasonic transducer (FUT). This class of transducer is a unimorph, but distinct from PMUT, CMUT, or flextensional configurations, with its traditional form commonly used for ultrasonic flow measurement and proximity sensing in industrial or automotive applications [9,10]. Most applications that utilize FUTs operate at ambient atmospheric pressure. However, there is industrial demand for ultrasonic transducers capable of the transmission and reception of ultrasound in higher pressure fluid environments, such as those encountered in petrochemical or gas metering [11,12,13,14]. The FUT consists of a circular membrane on to which a piezoelectric ceramic disc is bonded to the internal side of the membrane, typically by using an epoxy resin. The device is usually hermetically sealed using a backing material such as silicone in commercial configurations. The membrane itself is supported by a side-wall, which creates a boundary condition on the membrane akin to circumferential edge-clamping. As the piezoelectric ceramic disc is activated by a time-varying voltage, flexural modes are induced in the membrane. These modes of vibration can be estimated by mathematical theory of the resonant modes of the membrane [15], significantly expediting transducer design. The FUT can be fabricated from materials resilient to different types of fluid, making it an attractive transducer class for deployment in a range of different environments. The generalized schematic of the FUT is shown in Figure 1.

The compliance of the FUT’s flexing membrane makes it an efficient ultrasonic generator or detector. Ultrasonic transducers that operate primarily through the thickness or radial vibration modes of a piezoelectric disc generally require some form of impedance matching the front face [16]. In a FUT, the bending membrane is the component that directly couples with the environmental fluid.

Significant research has been undertaken to investigate the dynamic performance of the FUT in air-coupled environments [17,18,19,20]. These studies have provided a comprehensive analysis of how the FUT can be efficiently operated at high frequencies into the hundreds of kHz in different axisymmetric modes of vibration, and have reported the nonlinearity inherent in the dynamic response of the FUT, produced by a range of factors including changes in excitation conditions. These investigations have considered the dynamic performance of the FUT in atmospheric pressure conditions. However, the successful application of the FUT in environments with significant pressure fluctuations depends on the development of robust strategies for the design of FUTs appropriate for such conditions, and the reliable measurement of ultrasound in these environments.

In this study, the dynamic performance of different FUTs in air at elevated pressures is demonstrated. A custom pressure chamber is used to accommodate two FUTs, one for transmission of ultrasound and the other for detection. Experimental techniques including electrical impedance analysis and pitch-catch ultrasound measurement are utilized to show how the dynamic characteristics of FUTs can be characterized and monitored as functions of environmental pressure level. As an integral part of this study, principles of ultrasound measurement in air at elevated levels of pressure are investigated, where the influence of the physical characteristics of the pressure chamber on the reliability of measurement is assessed. It is anticipated that this research will drive the development of ultrasonic measurement technologies for applications characterized by pressure variation, such as those of the petrochemical, water, and gas metering industries.

## 2. Materials and Methods

### 2.1. Transducer Specifications

**Figure 2:**
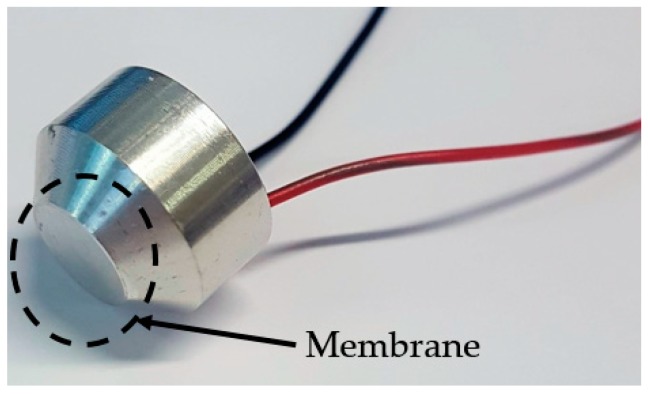
The flexural ultrasonic transducer (FUT) utilized in this study.

The principal objective of this research is to demonstrate the dynamics of FUTs in air at elevated pressure levels, realizing the potential of this technology for a wider range of applications, especially those vital to the petrochemical, gas, and water metering industries. A practical way of achieving this is to show the influence of elevated pressure levels on FUTs currently employed for industrial applications. An aluminium-capped FUT (Multicomp MCUST18A40B12RS, Farnell Components, UK) is used for this research. It is driven via a lead zirconate titanate piezoelectric ceramic disc bonded to the underside of its membrane (inside the FUT), and the transducer is resonant in the fundamental axisymmetric mode of vibration of the membrane around 40 ± 1 kHz. The transducer is shown in Figure 2.

The membrane of the transducer measures 10 ± 0.5 mm in diameter and the total height of the transducer is 12 ± 0.1 mm. It should be noted that the general profile of the cap differs from that shown in Figure 1, where there is a circumferential chamfer present in the transducer design used in this study. In general, cap designs can vary for different FUTs, and this can be for the control of boundary conditions or dynamic performance parameters such as resonance frequency. One advantage of the chamfer profile is that it allows the membrane of the FUT to be completely isolated from an applied support mechanism. Two forms of this transducer are analyzed in this research. The first generally conforms to the schematic shown in Figure 1, containing a silicone-type backing which creates a rear seal. For the purposes of this study, this configuration of transducer is referred to as the conventional flexural ultrasonic transducer (CFUT). The second type of transducer is an adapted form of this transducer where the seal is removed to permit equalization of pressure on both sides of the membrane, thereby eliminating any possible pressure imbalance between the internal structure of the transducer and the external environment. This is designated as a vented flexural ultrasonic transducer and given the nomenclature VFUT. Two VFUTs are used in this study which are the same design, VFUT_1_ and VFUT_2_, and all transducers are used interchangeably as generators or receivers of ultrasound. It is essential to demonstrate how different forms of FUT perform in a pressurized environment and the mechanisms by which measurement in a pressure chamber can be achieved. This will provide the necessary foundation for the development of more robust designs of FUT and ultrasound measurement systems for hostile environments.

### 2.2. Characterization Process

#### 2.2.1. Analysis of Resonance Characteristics

The first step of the characterization process is the measurement of the resonant vibration characteristics of each transducer. The resonance of a transducer can be identified through electrical impedance measurements and mode shape measurement. The mathematical theory demonstrating the derivation of the vibration modes of an edge-clamped plate, as shown by Leissa [15], can be considered as a fundamental thesis to the dynamic performance of the FUT, where the edge-clamped plate is analogous to the FUT membrane with a characteristically similar boundary condition. It should be noted that this analytical representation considers only the influence of the edge-clamping boundary condition on the vibration modes of a circular plate. The fundamental (0,0) mode of vibration can be simulated using this mathematical theory using the physical specifications of the transducer, such as those as shown in Section 2.1.

Experimental measurements of the (0,0) mode shapes are undertaken through laser Doppler vibrometry (Polytec OFV-5000) in air at atmospheric conditions of 1 bar and at room temperature, whereas the electrical properties are measured via an electrical impedance gain/phase analyzer (Agilent 4294A), comprising impedance Z and phase Φ. These are two properties of a transducer which are valuable in the analysis of transducer performance, particularly transducers subject to variations in boundary conditions or the pressure level of the environmental fluid. Measurements of Z and Φ are first made at atmospheric pressure (1 bar) and room temperature conditions. However, the change in resonance characteristics of the transducers can also be monitored via these properties as the environmental air pressure level is varied. For example, a local minimum in the impedance-frequency spectrum can be used to indicate a resonance frequency [21]. The frequency at which a specific minimum appears in the impedance-frequency spectrum can be tracked to show the change in the resonance frequency of a transducer as a function of environmental pressure level achieved as part of the wider characterization process, which encompasses electrical and pitch-catch ultrasound measurements. All data can be accessed as Supplementary Materials.

#### 2.2.2. Pitch-Catch Ultrasound Measurement

**Figure 3:**
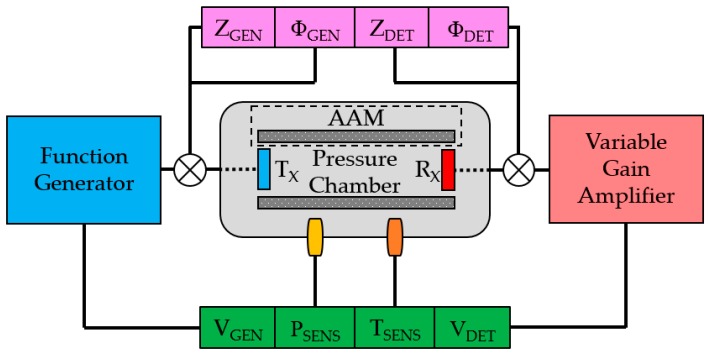
Overview of the experimental process, showing the parameters monitored during each experiment. The electrical characteristics of the transducers were measured in a step distinct from that of the pitch-catch ultrasound performance acquisition, and AAM refers to acoustically-absorbent material.

The resonance characteristics of the transducers can be measured through laser Doppler vibrometry and electrical impedance analysis. These techniques can be used first in atmospheric conditions at 1 bar and room temperature, as referred to in Section 2.2.1, but the measurement of the change in the dynamic performance of each transducer in response to air pressure variations must be performed inside a pressure chamber. A custom stainless steel chamber is used to house each FUT for pitch-catch ultrasound measurement. In this measurement configuration, one transducer is set as the transmitter of ultrasound $T_{X}$ and the other is designated as the receiver $R_{X}$. Sensor ports are incorporated into the pressure chamber to enable a range of parameters to be monitored as functions of environmental pressure level, comprising the electrical impedances of the generator transducer $Z_{GEN}$ and detector transducer $Z_{DET}$, the phases of the generator $\Phi_{GEN}$ and detector $\Phi_{DET}$, and the voltage magnitudes of the transmitted ultrasonic signal $V_{GEN}$ and the received signal $V_{DET}$. Pressurization of the chamber is achieved via a manual stirrup air pump and the environmental air pressure level $P_{SENS}$ is measured as a scaled voltage magnitude through a ratiometric pressure sensor (Honeywell) which is rated to above 200 bar, and the temperature inside the pressure chamber $T_{SENS}$ can be monitored using a thermocouple whose cabling is passed through a pressure-sealing gland in the pressure chamber. The experimental setup is shown in Figure 3.

Each transducer is positioned inside the pressure chamber, which comprises a cylindrical inner cavity. The cabling is passed through stainless steel pressure-sealing glands to the measurement instruments, and a function generator (Tektronix AFG3021B) is used to supply the $T_{X}$ transducer with a wideband sinusoidal signal burst of 2 cycles at 40 kHz, with a nominal peak-to-peak $V_{GEN}$ of 20 V_P-P_. A custom variable gain amplifier is used to boost $V_{DET}$, since the sensitivity of the FUT as a receiver can be relatively low compared to that of a calibrated instrument such as a capacitive microphone with its individual dedicated amplifier. Standard acoustic microphones are generally unsuitable in environments of elevated pressure. The variable gain amplifier contains a low-pass filter and possesses a −6 dB bandwidth of 29 kHz to 1.68 MHz. The electrical properties cannot be monitored simultaneously to pitch-catch ultrasound measurements, and so a switching system is administered to allow rapid and expedient transition between these measurements.

The pressure chamber incorporates acoustically-absorbent material (AAM) in the form of a foam-like structure which is circumferentially positioned around the internal side-wall of the pressure chamber to either absorb or scatter ultrasonic waves which are not propagated perpendicular to the membrane of the $R_{X}$ transducer. This is undertaken to reduce interference of the measured ultrasonic signal by reflections from the internal side-walls of the pressure chamber and peripheral internal structures such as the transducer supports. These supports are also shielded with AAM, but interference inside a metallic pressure chamber is difficult to avoid entirely, and so attempts to mitigate the influence of significant reflections are necessary. The AAM is folded to generate scattering and absorption of ultrasonic waves which are not propagated collinearly between the transducers. The internal bore diameter of the pressure chamber is 98 ± 0.25 mm and the longitudinal distance between the inside faces of the removable panels is 304 ± 0.25 mm. This constitutes a relatively small space in which the ultrasonic waves propagate, and so despite the presence of AAM in the internal cavity of the pressure chamber, all reflected ultrasonic waves will not be completely scattered or absorbed by the AAM during an experiment. Ultrasonic wave interference is very difficult to avoid altogether, but strategies such as that described in this section help to alleviate the disruptive influence of more significant interference and enable ultrasound measurements to be acquired with a satisfactory level of reliability. All data can be accessed as Supplementary Materials.

## 3. Results

### 3.1. Resonance Characteristics

**Figure 4:**
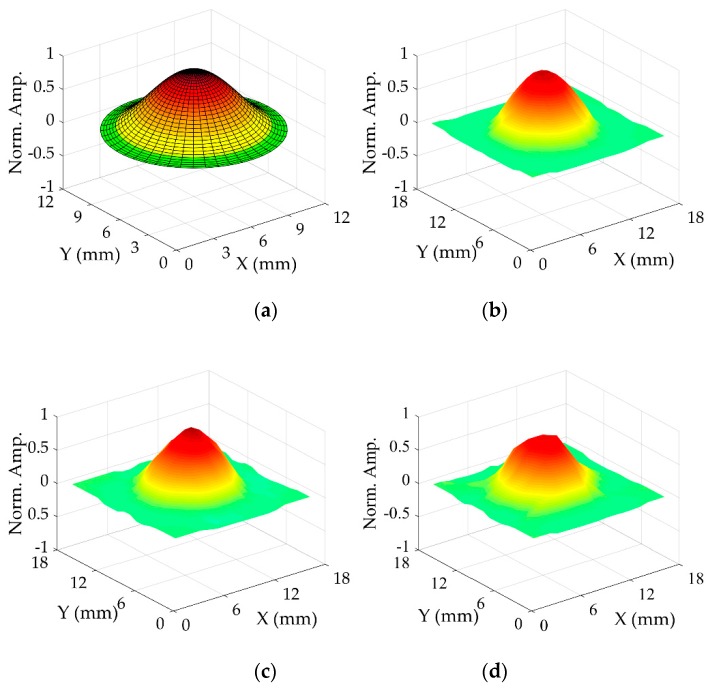
Mode shapes of each transducer displaying the fundamental (0,0) mode: (**a**) mathematically represented; and through laser Doppler vibrometry: (**b**) the CFUT; (**c**) VFUT_1_; (**d**) VFUT_2_. The vibration amplitudes of each mode shape have been individually normalized.

The mode shapes of the three transducers CFUT, VFUT_1_, and VFUT_2_ which are measured using laser Doppler vibrometry are shown in Figure 4, accompanied by the analytically-simulated mode shape which can be generated by using the fundamental relationships proposed by Leissa [15]. Each experimental mode shape can be observed to conform to the axisymmetric shape of the (0,0) mode as predicted by the mathematical simulation. The (0,0) mode of the CFUT was measured at 40.0 kHz, 39.6 kHz for VFUT_1_, and 40.4 kHz for VFUT_2_. Therefore, the resonance frequencies of the three transducers all exist around the transducer’s nominal resonance frequency of 40 ± 1 kHz as specified in Section 2.1. The variation in the fundamental resonance frequency between different transducers of the same nominal type can depend on several factors, some of which have already been reported [17,18,19,20]. Firstly, even minor sub-millimeter variations in the geometry of the transducer, for example, relating to membrane thickness or diameter, can cause significant differences in resonance frequency when compared with a nominally identical transducer. These variations in physical characteristics can become even more prominent if the influence of components, such as the piezoelectric ceramic disc is taken into consideration, since the FUT is a more complex system than that represented by the analytical simulation as shown in Figure 4a, which exclusively considers the vibration of the membrane. A marginal deviation of the piezoelectric ceramic away from the central-normal axis of the membrane compared to that of another FUT will produce a difference in resonance frequency. A further critical consideration is the boundary condition influence on the dynamic performance of the transducer. The analytical simulation displayed in Figure 4a exhibits an idealized edge-clamped boundary condition. This is unrealistic in practice, as the method by which the transducer is supported during operation directly affects the magnitude of the edge-clamping force on the membrane, and it is not always straightforward to ensure this force is evenly distributed around the circumference of the membrane or the cap. This is difficult to quantify but it is important to consider. The differences between the resonance frequencies measured through laser Doppler vibrometry will all be affected by the boundary conditions applied to each transducer. Although minor variations in the force on the membrane of each transducer could not be accurately monitored, the boundary condition was nominally equal for all mode shape measurements by using the same transducer holder fabricated from the amorphous thermoplastic polymer acrylonitrile butadiene styrene (ABS).

The assembly of the VFUT configuration differs marginally to that of the CFUT through the removal of the rear silicone-type seal. However, it is notable that there is only a minor variation in resonance frequency between the three transducers despite the absence of this rear seal component in the two VFUTs. This demonstrates the dominance of the membrane dynamics on the resonant performance of the FUT, and is a promising outcome in terms of the adaption of conventional FUTs and how they could be integrated into environments of varying pressure level. Even nominally identical transducers exhibit differences in dynamic performance as demonstrated in prior research [17,18,19,20,22]. However, there is an evident difference between the mode shapes measured for the VFUTs and the mode shape identified for the CFUT. There is a higher level of uniformity in the (0,0) mode shape associated with the CFUT, which is not observed for either VFUT. The fabrication of the VFUTs involved the attachment of an electrode connection to the metallic side-wall of the cap, due to the absence of any backing material through which a conventional electrode connection can typically be passed. There is, hence, a natural physical constraint placed on the VFUT structure via the side-wall which is not uniform, and therefore, it is likely that this has affected the uniformity of the mode shape. Since this has affected the (0,0) mode shape of both VFUTs, this is a likely cause.

**Figure 5:**
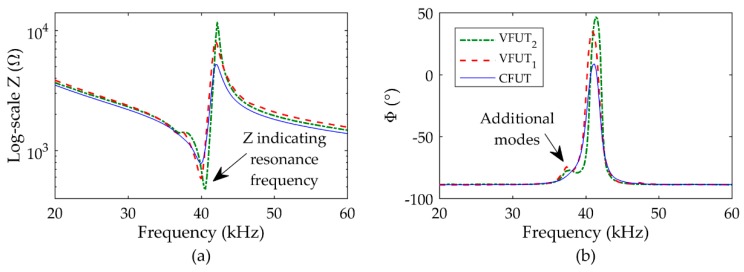
Electrical characteristics as functions of frequency for the transducers at standard room pressure (1 bar) and temperature: (**a**) electrical impedance; (**b**) phase.

The mode shape measurements were used as an indicator of resonance frequency which can be identified through electrical impedance analysis. The boundary condition on the transducer in the electrical impedance analysis was nominally similar to that applied in the mode shape measurements. However, the boundary condition on the holder is marginally different inside the pressure chamber compared to the external environment used for the mode shape measurements, since there is a force acting on the circumference of the ABS holder when secured in place. Furthermore, the excitation energy applied in the mode shape measurement was nominally 20 V_P-P_, but lower for the electrical impedance analysis in the order of 0.50 V_RMS_. The relationship between excitation amplitude and resonance frequency through dynamic nonlinearity has been reported [20,22] and must also be considered as a contributing factor to the discrepancy of resonance frequency using different experimental techniques. The Z and Φ spectra measured at standard room pressure and temperature conditions are shown in Figure 5 for the CFUT, VFUT_1_, and VFUT_2_.

The resonance frequency of each transducer can be measured through electrical impedance analysis by determining the frequency associated with the local minimum Z around resonance [21]. It should be noted that the Φ angle associated with resonance and a local minimum of Z is not necessarily coincident with a peak in the Φ spectrum. The results show the fundamental resonance frequency for the CFUT to be 40.00 kHz, 40.00 kHz for VFUT_1_, and 40.60 kHz for VFUT_2_. Each measured resonance frequency correlates closely to that observed through the mode shape measurement, broadly consistent with the specifications of the transducers and physical phenomena observed in prior research relating to the change in resonance with excitation [20,22]. Marginal discrepancies in resonance frequency between those measured via laser Doppler vibrometry and electrical impedance analysis can be attributed to differences in the clamping or boundary conditions required for each measurement technique [19], where it is known that the FUT is sensitive to even minor changes in the boundary condition. The Z magnitudes for the CFUT, VFUT_1_, and VFUT_2_ were measured to be 776.12 Ω, 582.90 Ω, and 479.33 Ω, respectively, and the corresponding Φ magnitudes were determined to be −38.18°, −21.16°, and −9.38°, respectively. There appears to be a greater discrepancy between the CFUT and the VFUT devices through the measurement of Z, and this may be in part a symptom of the silicone-type seal in the CFUT which is eliminated from the VFUT design.

**Figure 6:**
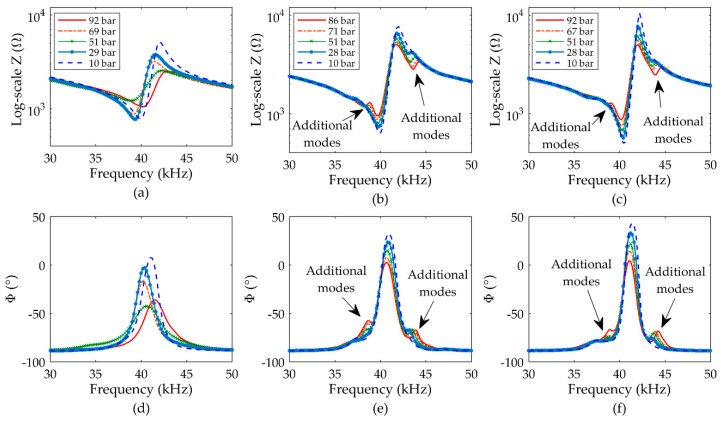
Electrical spectra of the transducers at different air pressure levels (± 0.5 bar) showing: (**a**) Z -Frequency (CFUT); (**b**) Z -Frequency (VFUT_1_); (**c**) Z -Frequency (VFUT_2_); (**d**) Φ -Frequency (CFUT); (**e**) Φ -Frequency (VFUT_1_); (**f**) Φ -Frequency (VFUT_2_).

Additionally, the Z and Φ spectra both show additional modes preceding that of the fundamental mode. These modes can be attributed to a vibration energy transfer through the ABS holder, and are more prominent for both VFUT devices, which is expected since the side-wall of the VFUT cap is more compliant than that of the CFUT as it lacks the silicone seal component. The additional modes also occur at the same frequency for both VFUT transducers, thus providing further evidence that it must be as a consequence of a common factor between both measurements, which in this case is the ABS holder. The Z and Φ measurements shown in Figure 5 can be used as a baseline to monitor the Z and Φ spectra as the air pressure level within the chamber is raised from 1 bar. The results for the three transducers from an air pressure level inside the pressure chamber of 10 bar towards a nominal pressure level of 90 bar are shown in Figure 6, and the magnitudes of Z, Φ, and resonance frequency ($f_{R}$) changes as functions of air pressure level are summarized in Table 1.

The Z spectra clearly show a resonance instability for the CFUT compared to either VFUT, which can be attributed to the influence of air pressure imbalance between the inside of the CFUT and the external pressurized environment on the transducer membrane. The seal of the transducer deforms, thus causing relatively significant shifts in resonance which are undesirable in practical application. This does not occur in either VFUT since the pressure on either side of the membrane is balanced, and consequently, the resonance frequencies of the VFUTs are comparably stable towards 90 bar. As the air pressure level is increased, the Z of the CFUT does not exhibit a consistent increase or decrease but instead is unstable, comparable to the trend of resonance frequency. In contrast, the Z of each VFUT steadily increases as the air pressure level rises. One explanation for this is that as the air pressure inside the pressure chamber increases, there is consequently a greater pressure acting on both the membrane and the piezoelectric ceramic disc of each VFUT, thereby generating a higher characteristic Z. This also explains why there are minor changes to resonance frequency for both VFUTs, where the influence of higher air pressure on the piezoelectric ceramic disc must be considered. The reason that a monotonic change in Z is not evident in the electromechanical performance of the CFUT is that the silicone-type seal component deforms in response to different air pressure levels, and so the influence of air pressure level on the membrane and piezoelectric ceramic disc of the CFUT is difficult to predict. In terms of practical application, the stabilities of both $f_{R}$ and Z are important, and this demonstrates the unsuitability of the CFUT for operation at elevated pressure levels. There is also a relatively high risk of leaks forming in the CFUT, which will also lead to dynamic instability or unreliability. These limitations associated with the CFUT necessitate the development of alternative configurations of the transducer, such as the VFUT.

It is of interest to note the dissimilarity between the relationship of Z and pressure level, and Φ and pressure level. In the impedance spectra of the VFUTs shown in Figure 6, a relatively monotonic increase in Z has been observed, but this is not observed for Φ. There is an interdependency between Z, $f_{R}$, Φ, and pressure level. Although these characteristics should all be monitored in the operation of ultrasonic transducers subjected to varying levels of environmental pressure, it is essential to understand that different levels of pressure affect Z and Φ differently, and this is important to consider in the development of more complex ultrasonic measurement systems, for example, those utilizing multiple elements of FUT. A further behavior to consider for the results shown in Figure 6 relates to the emergence of additional modes around that of the fundamental mode as the air pressure level changes. While unstable in terms of Z, Φ, and $f_{R}$, the Z and Φ spectra for the CFUT do not show the emergence of any other artefacts. This is not the case for either of the VFUTs, where additional modes can be observed to emerge at frequencies either side of the fundamental mode $f_{R}$. These additional modes begin to appear even at relatively low air pressure levels around 10 bar and become more prominent as the air pressure level increases. Despite this, the $f_{R}$ of each VFUT remains relatively stable. Therefore, these additional modes are likely caused by the influence of the rising air pressure level on the boundary condition of each VFUT, for example, through the ABS holder. This change in the boundary condition will affect the electrical characteristics of the transducers and is more noticeable for the VFUT compared to the CFUT since it is structurally more compliant, as referred to earlier in this Section. However, it is possible that the emergence of these additional modes could be mitigated in an optimized form of the VFUT design, for example, through significantly increasing the mass of the cap side-wall. This opens up an area of future research in the development of FUTs for operation at elevated pressure levels.

### 3.2. Pitch-Catch Ultrasound Measurement

#### 3.2.1. Influence of Air Pressure Level on Voltage Response

**Figure 7:**
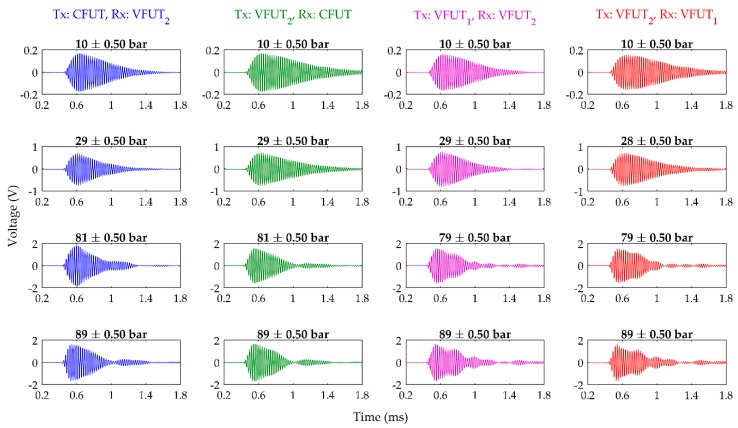
A-scans for increasing levels of air pressure inside the pressure chamber utilizing different combinations of $T_{X}$ and $R_{X}$ transducers.

The voltage signals for the FUTs in different combinations of $T_{X}$ and $R_{X}$ can show the effectiveness by which an accurate ultrasound measurement can be conducted between a pair of FUTs. This method is desirable because the transducer nominated as the $T_{X}$ can be efficiently switched to $R_{X}$ and vice-versa. This can be undertaken in applications such as ultrasonic flow measurement, where the upstream and downstream measurements of flow are different due to the influence of fluid velocity on the accuracy of the ultrasonic wave measurement. In this section, voltage-time (A-scan) signals are presented for different levels of environmental air pressure and for four different pitch-catch configurations of $T_{X}$ and $R_{X}$. Through these measurements, a $T_{X}$-$R_{X}$ system consisting of a CFUT is directly compared with one incorporating two VFUTs. The results of the pitch-catch measurements using the experimental specifications shown in Section 2.2.2 are exhibited in Figure 7 for four different air pressure levels inside the pressure chamber. The measurements recorded at these pressure levels are a selection of experimental results that represent extremes of air pressure level inside the pressure chamber.

There is not a noticeable difference between the A-scans around a nominal pressure level of 10 bar. No discernable interference or additional ultrasonic waves have been detected, and in terms of practical application, each signal is representative of a clear and reliable ultrasound measurement. As the pressure level is raised towards 30 bar, the signals remain characteristic of A-scans obtained from a single-frequency ultrasonic wave, although the resonant decay of the pitch-catch configuration with the CFUT as the $T_{X}$ is noticeably shorter in time. This is likely a symptom of the larger pressure imbalance between both sides of the transducer membrane causing a greater physical influence on the transducer itself compared to either of the VFUTs. One reason a comparable effect is not detected when the CFUT is operated as $R_{X}$ is because the type of FUT used in this study is significantly more effective as a transmitter of ultrasound than a receiver, despite the inclusion of a variable gain amplifier in the experimental setup. As a transmitter, the piezoelectric ceramic disc directly induces the vibration motion of the compliant membrane. However, as a receiver, the relative magnitudes of the ultrasonic waves which interact with the membrane of the FUT must overcome the relatively high inertia of the membrane and piezoelectric ceramic disc in order to produce a voltage signal in the piezoelectric ceramic disc. Therefore, amplification is usually required for a $R_{X}$ transducer. In general, the displacement of the FUT operating as the transmitter is significantly higher than that of the receiver, and therefore, the transmitter will experience a stronger damping effect due to the imbalance of air pressure between the internal structure of the transducer and the external environment. This will have a prominent effect on the dynamics of the CFUT since there is a pressure imbalance caused by the seal.

The key change in characteristic behavior for all the FUT configurations shown in Figure 7 can be observed as the pressure level passes 80 bar. In each A-scan, there are additional peaks evident which can be considered as reflections that can be associated with structures inside the pressure chamber, including the internal side-wall and the surfaces of the removable panels at either end of the pressure chamber. As the environmental pressure level is raised, the acoustic properties of the air will also change, directly influencing the nature of ultrasonic wave propagation inside the pressure chamber. It is also of interest that the emergence of ultrasonic wave interference is only conspicuous in each A-scan for any $T_{X}$ and $R_{X}$ transducer combination at a pressure level above approximately 30 bar. This is a relatively high-pressure level for many conventional ultrasound measurement applications. It is therefore essential to understand the influence of elevated pressure levels on the characteristic dynamic response expected from a combination of $T_{X}$ and $R_{X}$ transducers for reliable measurement. Considering all signals together, it is possible that these additional ultrasonic waves or reflections are embedded in each signal shown in Figure 7, even those at 10 bar. It is only as the pressure level is increased, that these peaks become distinguishable as air damping increases.

The fact that there is an increased number of reflections or additional ultrasonic waves detected for the VFUTs is to be expected, since the VFUT does not contain a component which can act as a damper or increase the effective mass of the transducer. It is of particular interest to note the signal where VFUT_2_ is $T_{X}$ and the CFUT is $R_{X}$ is showing the clear influence of the CFUT in the overall measured response signal, where there are fewer additional ultrasonic waves compared to the two VFUTs as $T_{X}$ and $R_{X}$. As the pressure level is raised towards 90 bar, similar A-scans are exhibited as those around 80 bar for the two VFUTs but with marginally higher amplitudes. However, the characteristic A-scan for the CFUT as $T_{X}$ is noticeably different from that at 80 bar. In general, the A-scan for the CFUT does not appear to be consistent as the pressure increases, and the influence of a further ultrasonic wave can be seen to emerge, as shown in the signal for the CFUT as $T_{X}$ at 90 bar. A clear advantage of the VFUT in terms of pitch-catch measurement is that they are effectively interchangeable as $T_{X}$ and $R_{X}$ transducers, since the A-scans appear very similar, irrespective of the $T_{X}$ and $R_{X}$ configuration. This is particularly significant because although the two VFUTs do not possess identical resonance frequencies, which is a common phenomenon for FUTs, the results show that utilizing two VFUTs with dissimilar resonance frequencies would be feasible for practical application. The resonance frequencies of FUTs specified to be identical have been known to vary in the order of hundreds of Hz. However, this interchangeability is not observed in the A-scans obtained from the pitch-catch configuration involving the CFUT, particularly at higher levels of pressure such as around 80–90 bar. The importance of considering the electrical impedance measurements together with the pitch-catch measurements is evident for a reliable ultrasound measurement system at elevated pressure levels, where it is vital to be able to accurately characterize the dynamic performance of each transducer.

**Figure 8:**
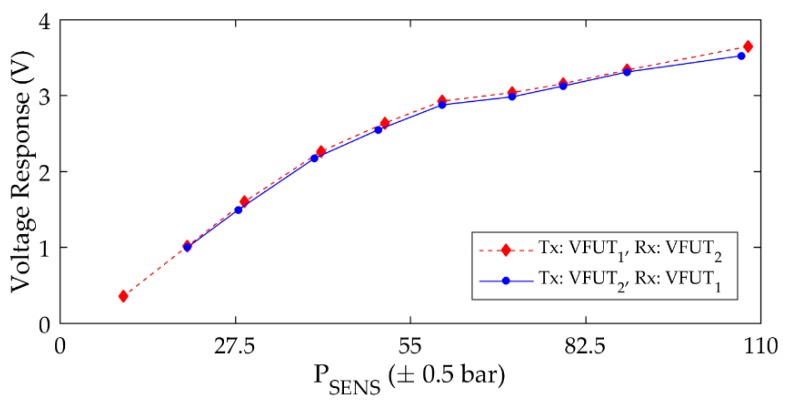
The voltage response as a function of air pressure level for VFUT_1_ and VFUT_2_ used alternately as $T_{X}$ and $R_{X}$ transducers. Note the (peak-to-peak) voltage response in each case is extracted from the first peak in the A-scan.

In contrast to the performance of the CFUT, the VFUT exhibits an improved level of interchangeability which can be clearly demonstrated if the results of Figure 7 are compared with those shown in Figure 8, which represent the peak-to-peak voltage magnitudes of the first peaks in the A-scans in each case for VFUT_1_ and VFUT_2_ towards 100 bar.

It should be noted that the voltage response magnitudes in Figure 8 are directly extracted from ultrasound measurement data, and the influence of ultrasonic wave interference is not considered. The increase in voltage response as a function of pressure for these transducers can be attributed to the rise in the mass loading on the transducer membrane, where the boundary conditions of each transducer are directly affected. In general, this experimental result illustrates the interchangeability of two FUTs designed for operation in environments of elevated pressure. Although the influence of any ultrasonic wave interference is not removed through signal processing, the ability to generate and detect ultrasonic waves with similar amplitudes irrespective of the $T_{X}$ and $R_{X}$ combination is an important outcome for ultrasound measurement with FUTs at elevated pressure levels. The implementation of a burst excitation signal with a low number of cycles in the experimental process is also significant in this context. This form of signal is wideband and is appropriate for use with a combination of FUTs with marginally different resonance frequencies. An excitation signal with a high number of cycles will result in a narrow bandwidth of the generated ultrasonic wave in air. Future research can focus on excitation signal optimization for ultrasound measurement in environments of relatively small size, such as the pressure chamber used in this study.

**Figure 9:**
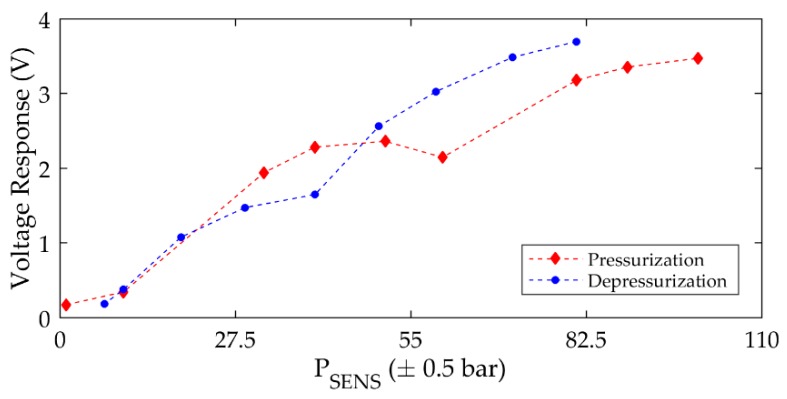
The hysteresis associated with the voltage response as a function of air pressure level for the CFUT as the $T_{X}$ transducer and VFUT_2_ as the $R_{X}$ transducer. Note the (peak-to-peak) voltage response in each case is extracted from the first peak in the A-scan.

Ultrasound measurement applications at elevated pressure levels, including gas and water metering as referred to in Section 1, will involve fluctuations of pressure level. It is important to understand how these pressure fluctuations can influence ultrasound measurement. To analyze this phenomenon, the environmental air pressure is increased towards 100 bar and then decreased. The $T_{X}$ and $R_{X}$ transducers are arbitrarily selected as the CFUT and VFUT_2_, and the peak voltage magnitude of the first peak in the A-scan is monitored as the environmental air pressure level is modulated. The results are shown in Figure 9.

The results can be linked to those shown in Figure 8 and show a clear hysteresis in the voltage response. There is a similarity between the data sets at relatively low-pressure levels, in particular below approximately 30 bar, but a distinct deviation emerges above this pressure level. There is a decrease in voltage response close to 60 bar in the pressurization step and a similar discontinuity is evident in the depressurization step, but at a different environmental pressure level closer to 40 bar. This behavior can be exclusively attributed to the dynamic performance of the CFUT. In terms of the underlying physics, it is likely that the decrease in voltage response during the pressurization step is associated with a leak forming in the CFUT. Following a leak formation, the pressure inside the CFUT would begin to balance with the pressure of the external environment. During the depressurization step, the pressure would, therefore, reduce to such a level that the silicone seal of the CFUT deforms and creates a sealing effect once more, and the pressure level inside the CFUT would, therefore, be out of balance with the pressure level of the external environment from this stage. In general, the CFUT exhibits leakage above a threshold level of environmental air pressure, but the pressure inside the CFUT may not entirely reach that of the environment before it reseals. The CFUT can then exhibit leakage from its internal cavity at a lower pressure than that of the air environment via the pressure differential which has been created.

The increase or decrease of environmental pressure level also induces changes in the boundary conditions of the transducers. This physical effect would impart a more critical influence on the dynamic performance of the CFUT due to the relative flexibility of the silicone-type seal. It should also be noted that it would be conceivable that as a transducer is exposed to higher levels of pressure, there may be structural deformation induced in the transducer which is non-reversible, and that could create a hysteretic response such as that shown in Figure 9. This phenomenon is important to consider for the practical application of FUTs, especially those incorporating seals and if the amplitude of vibration is a critical performance parameter.

#### 3.2.2. Analysis of the Resonant Ring-Down Response

**Figure 10:**
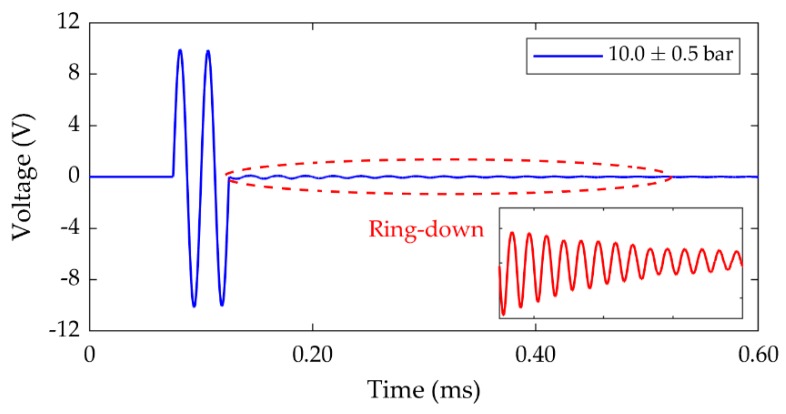
A typical example of a $T_{X}$ signal used in this study, showing the resonant ring-down phenomenon in the signal immediately after the cessation of the cycle burst. The ring-down is scaled for clarity shown by the inset figure.

Up to this stage, the analysis of the FUTs has utilized two strategies. First, the electrical characterization of the transducers to define the frequency window in which the ultrasonic waves can be detected by the transducer configured as $R_{X}$, and second, the dynamic performance of different combinations of $T_{X}$ and $R_{X}$ transducers, where the A-scans as shown in Figure 7 demonstrate the dynamic performance of the entire $T_{X}$ and $R_{X}$ system at different levels of air pressure. This analysis can be extended to consider the influence of air pressure level on the dynamic characteristics of the FUT configured as the $T_{X}$. transducer upon the termination of the excitation signal. The typical form of the 2-cycle, 40 kHz, 20 V_P-P_ sinusoidal burst signal used in this study as the input to the $T_{X}$ transducer is shown in Figure 10.

**Figure 11:**
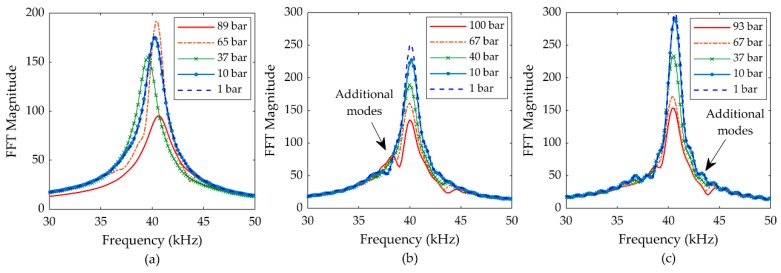
FFTs of the resonant ring-down decay region of the $T_{X}$ signals associated with the transducers at different air pressure levels (± 0.5 bar): (**a**) CFUT; (**b**) VFUT_1_; (**c**) VFUT_2_. Note the difference in the ordinate axis scales between the CFUT and the two VFUTs.

This signal was measured from the function generator directly on the oscilloscope. There is a dynamic phenomenon observable in the excitation signal as shown in Figure 10, which can be attributed to the decaying resonant vibration of the transducer membrane after the excitation signal is terminated. This phenomenon can be exploited as a practical and rapid estimator of FUT resonance [18] for any FUT. In Figure 10, the ring-down phenomenon has been scaled for clarity to show a typical form of resonant decay in the $T_{X}$ signal of a FUT. Here, the measurement of this phenomenon is particularly useful for investigating the influence of air pressure on the dynamic performance of the FUT. The fast Fourier transform (FFT) of the ring-down signal for each transducer as a function of environmental air pressure level is shown in Figure 11 for the CFUT, VFUT_1_, and VFUT_2_.

The results show a correlation between the response of the electrical characteristics of the transducers measured through electrical impedance analysis and changes in the environmental air pressure as shown in Figure 6. Note there is a relatively large variation in the resonant frequency of the CFUT, which is not observed for either VFUT. Furthermore, additional modes have also been detected in the responses of both VFUTs, which can be attributed to the influence of the ABS supports on the physical resonance, but also must be a consequence of pressure change on the boundary conditions of the transducers, since the influence of this phenomenon changes as a function of pressure. The VFUTs are more susceptible to changes in environmental pressure level since their side-walls are more compliant than that of the CFUT, and is the reason the influence of any additional modes is less prominent in Figure 11a. As the air pressure is increased, the peaks in the FFT magnitudes steadily reduce for the VFUTs, but this is not observed for the CFUT. The monotonic reduction in FFT amplitude constitutes a pattern of behavior which could potentially be predicted with reasonable accuracy in more complex ultrasound measurement systems in environments of varying pressure level. However, due to the presence of a silicone-type seal, no such performance estimations could be reliably established for the CFUT. It is clear that the changing level of air pressure directly affects the fundamental dynamic properties of FUT irrespective of configuration, but that transducer designs such as the VFUT may be more suitable for complex ultrasound measurement systems. The CFUT utilized in this study remains operational even towards 90 bar of environmental pressure, but there is little evidence it could be operated with reliability, and the CFUT design has been observed to fail at lower levels of environmental pressure.

The experimental method and the results demonstrated in this study have shown the important factors which must be considered in the implementation of FUTs for the measurement of ultrasound at elevated levels of air pressure. The techniques by which the properties of the transducers have been measured and the characteristics of the pressurized environment controlled can be extended to alternative configurations of ultrasonic transducer. These techniques will be invaluable in the development of more robust ultrasound measurement systems for a wide range of industrial applications. The mitigation of ultrasonic wave interference is essential and will require the study of fluid physics in the pressure chamber at elevated pressure levels. The influence of temperature on the performance of the FUT also requires investigation. The outcomes of this research can be used to optimize FUTs specifically for operation at elevated pressure levels and in fluid environments encompassing different gases and liquids.

## 4. Conclusions

This study has demonstrated the influence of air pressure in the transmission and measurement of ultrasonic waves in a metallic pressure chamber using FUTs. Three FUTs have been studied comprising two configurations: a commercial-type aluminium type, and a transducer modified specifically for operation at elevated pressure levels by ensuring a balanced pressure between the internal structure of the transducer and the external environment. Principles of measurement have been reported, including the demonstration of a novel experimental process comprising an electrical impedance analysis and pitch-catch ultrasound measurement with simultaneous control and measurement of environmental pressure level. The dynamic characteristics of the three transducers were measured before they were subjected to elevations in environmental air pressure inside a custom pressure chamber. The dynamic performance of the transducers in these conditions was measured in different combinations of transmitter and receiver. The results show the ability of the FUT to operate effectively as either a transmitter or receiver of ultrasound in an air environment of elevated pressure level towards 100 bar using a relatively wideband excitation signal. Although the CFUT is operational even at pressure levels towards 100 bar, its frequency response is less stable than is observed with the VFUTs in terms of both electrical and dynamic performance. The VFUT is more suitable for reliable ultrasound measurement at elevated or changing pressures. However, the greater effective mass and damping of the sealed CFUT reduces the ringing of the membrane. It is anticipated that this fundamental research will generate new developments in the design and fabrication of FUTs for operation at elevated pressure and in different types of fluid. Future research will also focus on the optimization of the measurement environment to further mitigate the influence of ultrasonic wave interference and enable the reliable exploitation of different forms of excitation signal.

## 5. Patents

A patent has been filed, *Flexural ultrasonic transducer*, Application Number GB1811922.2.

## Figures and Tables

**Table 1:** Magnitudes of Z, Φ, and $f_{R}$ at different air pressure levels measured through electrical impedance analysis, where specific pressure levels can be correlated with the associated magnitudes shown in Figure 6.

Nom. $P_{SENS}$ (bar)	CFUT			VFUT_1_			VFUT_2_		
	Z (Ω)	Φ (°)	$f_{R}$ (kHz)	Z (Ω)	Φ (°)	$f_{R}$ (kHz)	Z (Ω)	Φ (°)	$f_{R}$ (kHz)
10	779.53	−35.68	40.00	625.67	−20.08	40.00	498.67	−34.49	40.40
30	767.65	−40.26	39.40	729.57	−32.65	39.80	562.72	−22.55	40.40
50	1212.85	−63.56	38.80	792.18	−28.64	39.80	678.32	−40.02	40.20
70	869.07	−49.84	39.20	872.84	−41.54	39.60	747.12	−38.33	40.20
90	1043.67	−59.25	40.20	946.63	−43.47	39.60	859.16	−38.49	40.20

## Supplementary Materials

The experimental data can be accessed at: http://www2.warwick.ac.uk/fac/sci/physics/research/ultra/research/PresMeas.zip; The project web-site with full details of the research programme can be accessed via the following link: https://warwick.ac.uk/fac/sci/physics/research/ultra/research/hiffut/.
